# Supplementary material for: Antibody and Cellular Immune Responses in Old α1,3-Galactosyltransferase-Knockout Mice Implanted with Bioprosthetic Heart Valve Tissues
Source: Bioengineering (Basel). 2025 Dec 31;13(1):53. doi: 10.3390/bioengineering13010053 (PMC12838042; doi:10.3390/bioengineering13010053)
Supplement: Supplementary file 1 [file bioengineering-13-00053-s001.zip › bioengineering-3983448-supplementary.pdf]

Supplementary Material

Antibody and Cellular Immune Responses in Old α1,3-Galactosyltransferase-Knockout Mice Implanted with Bioprosthetic Heart Valve Tissues

Kelly Casós, Roger Llatjós, Arnau Blasco-Lucas, Sebastián G. Kuguel, Fabrizio Sbraga, Cesare Galli, Vered Padler-Caravani, Thierry Le Tourneau, Marta Vadori, Jean-Christian Roussel, Tomaso Bottio, Emanuele Cozzi, Jean-Paul Soulillou, Manuel Galiñanes, Rafael Máñez and Cristina Costa*.

*Correspondence should be addressed to Cristina Costa.
Institut de Química i Biotecnologia de Barcelona (IQBB),
Consorci d'Educació de Barcelona (CEB),
Passeig de Salvat Papasseit, 5

08003 Barcelona, SPAIN

e-mail: ccosta_valles@yahoo.com

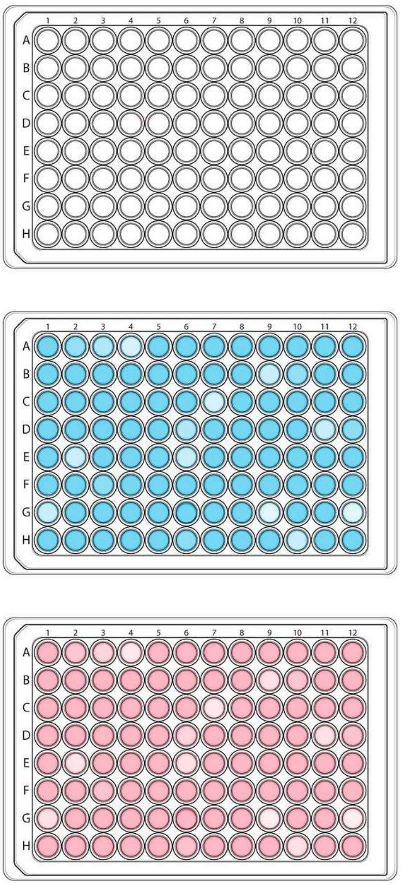

Plate 1: sera were first diluted 2x in PBS 1% BSA (2% for IgM, 1% for IgG) in a 96-well plate.

Plate 2: identically distributed 96-well plate/s containing an equal volume of diluent with GAS914 2x (1 mg/ml) or without for subsequent mixing with the sera for pre-incubation for 20-30 minutes at room temperature.

Plate 3: confluent PAEC were harvested in PBS 1% BSA and distributed in an equivalent layout. Supernatants were carefully aspirated and cells resuspended with the preincubated sera for 30-min incubation at 4°C followed by staining with 2ry Ab.

FACs

Supplementary Figure 1 (S1). The procedure for determination of serum anti-Gal and anti-non-Gal IgM and IgG reactivities by flow cytometry is explained here for further clarification of the protocol described in Materials and Methods. The same serum dilution was used for the determination of all reactivities by each serum sample. After testing various serum concentrations, we finally ruled out the possibility of conducting both determinations in a single assay and applied a 1% serum for measurements of IgM reactivity and 0,5% serum for IgG reactivity determinations. This 0,5% serum results in very low values of IgG reactivity but prevents interference and saturation of the system by anti-Gal IgM that can compete for the Gal antigen present on the PAEC surface. In the largest experiments (many mouse serum samples assessed simultaneously), IgG and IgM measurements were conducted in separate experiments/days.

For each flow cytometric experiment, all the sera were first diluted 2x in PBS 1% BSA (2% for IgM, 1% for IgG) in a 96-well plate with identified locations and subsequently

transferred to an identically distributed 96-well plate containing an equal volume of diluent with GAS914 2x (1 mg/ml) or not for incubation for 20-30 minutes at room temperature. During this time frame, confluent PAEC were harvested and washed twice with PBS 1% BSA by resuspension and centrifugation (one wash in conical tube and one in 96-well plate after equal distribution). After thorough elimination of supernatants by aspiration, PAEC were resuspended in the diluted serum and incubated for 30 minutes at 4°C. Standard procedures were next followed for incubation with secondary antibodies specific for either mouse IgM or mouse IgG, washed in PBS and transferred to FACs tubes to be passed through the Gallios flow cytometer (Beckman Coulter, Brea, CA, USA). The Mean fluorescence intensity (MFI) for each determination was obtained with an overlay histogram with Kaluza software (Beckman Coulter, Brea, CA, USA) as shown in the representative images (Suppl. Fig 2 and 3). The procedure can be escalated to more plates depending on the number of samples and ability of the operator.

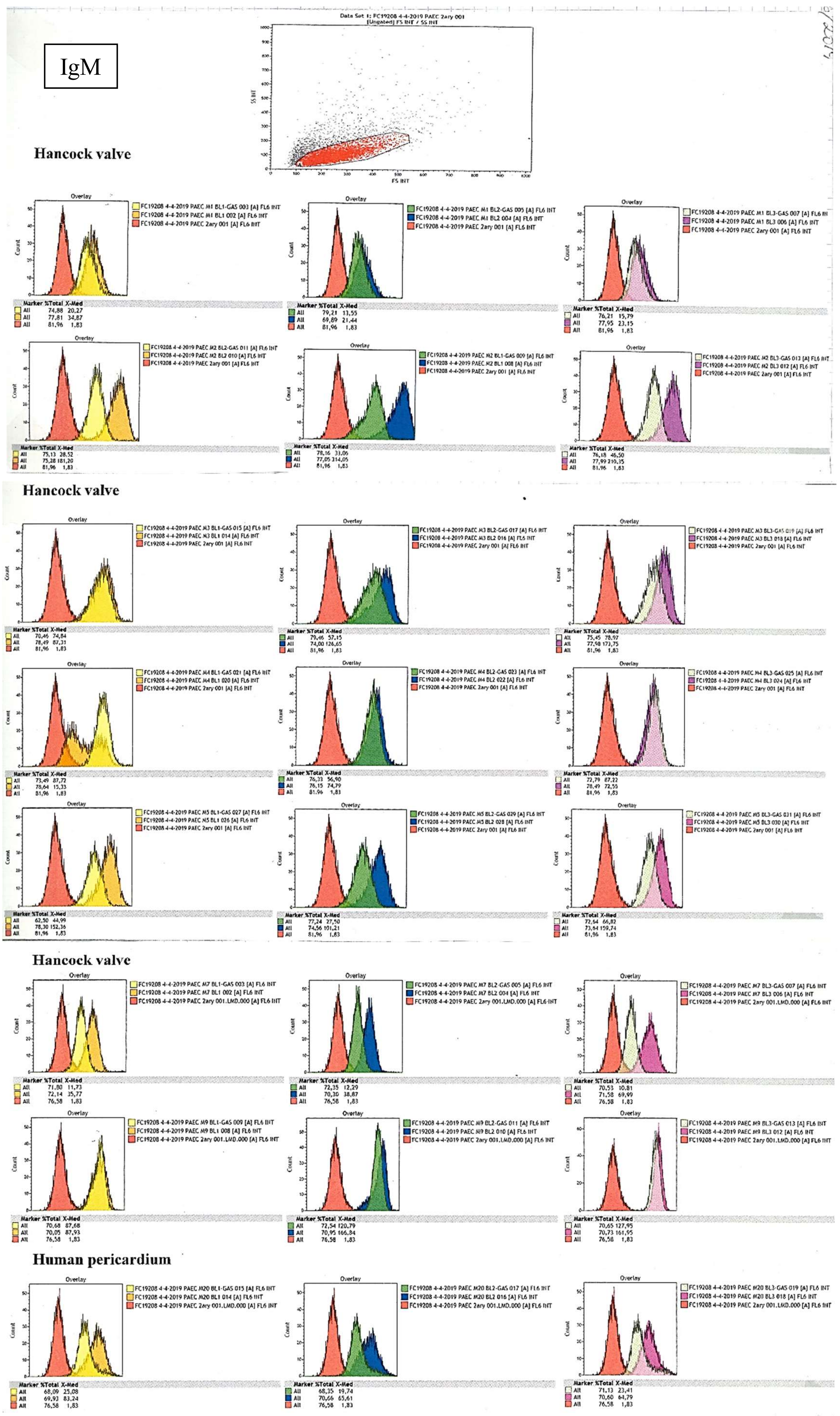
IgM
Hancock valve
Hancock valve
Hancock valve
Human pericardium

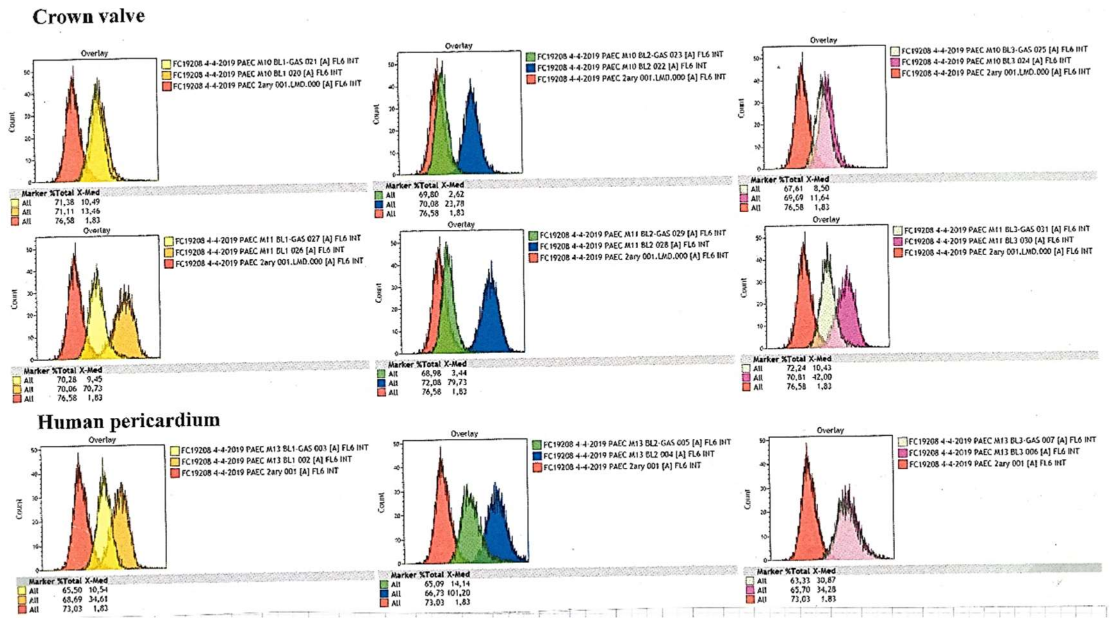

Supplementary Figure 2 (S2). Representative images of original flow cytometry data for determinations of serum anti-Gal and anti-non-Gal IgM reactivity (Mean fluoresce intensity) corresponding to adult Gal KO mice grafted subcutaneously with BHV tissues for 2 months. The procedure was conducted as described in Materials and Methods and Figure S1 using an Alexa Fluor 647–conjugated goat anti-mouse IgM secondary antibody in a Gallios flow cytometer with Kaluza software (Beckman Coulter, Brea, CA, USA). The only gate applied for the analysis is shown in red at the top of the figure (identified as “A”) that corresponds to a homogenous PAEC population.

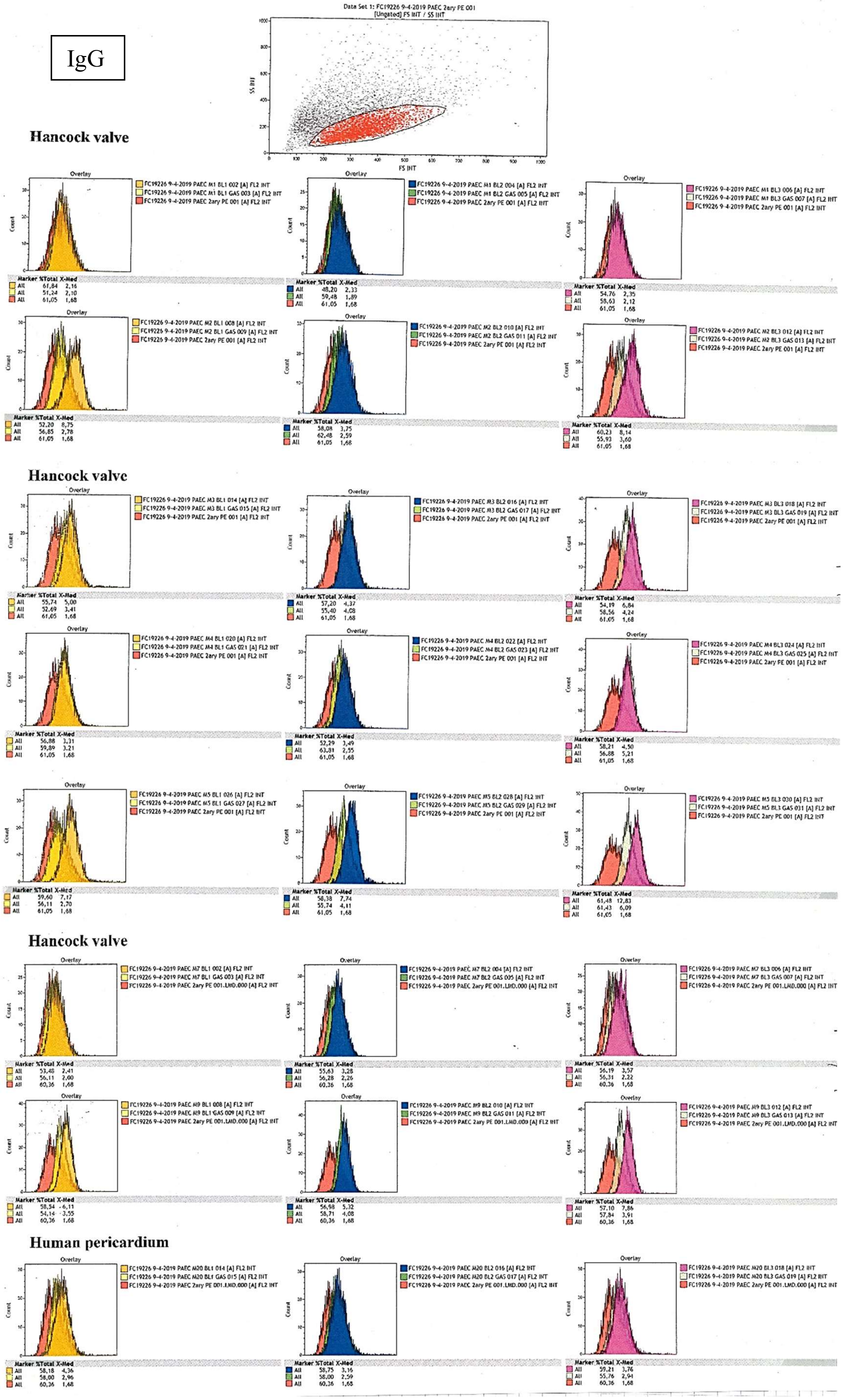
IgG
Hancock valve
Hancock valve
Hancock valve
Human pericardium

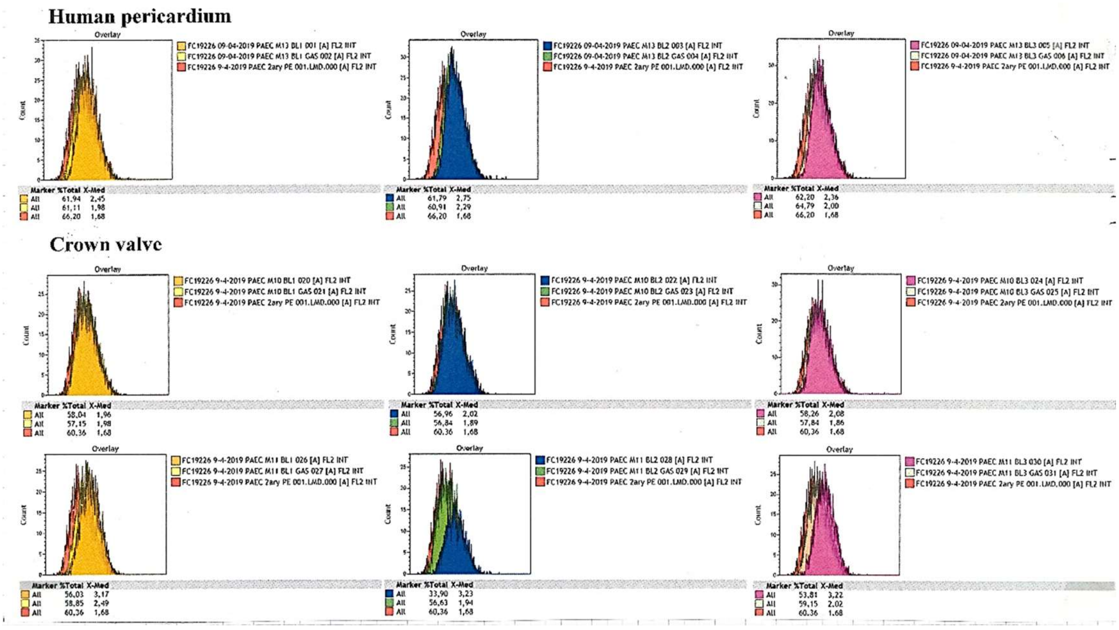

Supplementary Figure 3 (S3). Representative images of flow cytometry data for serum anti-Gal and anti-non-Gal IgG reactivity (Mean fluoresce intensity) corresponding to adult Gal KO mice grafted subcutaneously with BHV tissues for 2 months. The procedure was conducted as described in Materials and Methods and Figure S1 using a PE-conjugated goat anti-mouse IgG secondary antibody in a Gallios flow cytometer with Kaluza software (Beckman Coulter, Brea, CA, USA). The only gate applied for the analysis is shown in red at the top of the figure (identified as “A”) that corresponds to a homogenous PAEC population.
